# Therapeutic Potential of Engineered Extracellular Vesicles

**DOI:** 10.1208/s12248-018-0211-z

**Published:** 2018-03-15

**Authors:** Kyle I. Mentkowski, Jonathan D. Snitzer, Sarah Rusnak, Jennifer K. Lang

**Affiliations:** 1 Department of Medicine, Division of Cardiology, Jacobs School of Medicine and Biomedical Sciences, Clinical and Translational Research Center, 895 Ellicott Street, Buffalo, NY 14203, USA

**Keywords:** Apoptotic bodies, Engineered exosomes, Exosomes, Extracellular vesicles, Microvesicles

## Abstract

Extracellular vesicles (EVs) comprise a heterogeneous group of small membrane vesicles, including exosomes, which play a critical role in intracellular communication and regulation of numerous physiological processes in health and disease. Naturally released from virtually all cells, these vesicles contain an array of nucleic acids, lipids and proteins which they transfer to target cells within their local milieu and systemically. They have been proposed as a means of “cell-free, cell therapy” for cancer, immune disorders, and more recently cardiovascular disease. In addition, their unique properties of stability, biocompatibility, and low immunogenicity have prompted research into their potential as therapeutic delivery agents for drugs and small molecules. In this review, we aim to provide a comprehensive overview of the current understanding of extracellular vesicle biology as well as engineering strategies in play to improve their therapeutic potential.

## INTRODUCTION

The therapeutic potential of drugs and small molecules hinges on directed delivery to the site of injury while avoiding off target side effects. Numerous synthetic platforms, including polymeric nanoparticles and liposomes, have been investigated with only a small number successfully approved by the FDA (1). This gap in clinical translation is largely secondary to two significant obstacles: the inherent difficulty in overcoming our body’s ability to identify and remove foreign material, and the development of site-specific targeting mechanisms (2). While designing nanodelivery systems, we have often looked to nature as a source of inspiration, attempting to replicate surface marker expression, morphology, and attributes of biological carriers to enhance targeted delivery and avoid clearance from the circulation. In recent years however, the idea of *enhancing* rather than replicating biological carries, such as exosomes, has gained more attention as a feasible option for therapeutic delivery.

Extracellular vesicles were first observed by electron microscopy in the 1980s, but regarded as no more than cellular garbage bags for expired protein until the early 2000s, when they were discovered to contain and transfer functional RNA to target cells (3). They are now recognized as important and universal agents of intercellular communication, shuttling numerous signaling molecules, proteins, lipids, mRNA, miRNA, siRNA, IncRNA, and extra-chromosomal DNA throughout the circulatory system (4).

Exosomes, a nanosized subset of EVs which originate during the formation of multivesicular bodies (MVB), are secreted constitutively by fundamentally all cells in physiological conditions. Their production can be stimulated, and the contents of their cargo regulated by stress or disease. They possess intrinsic biological activity through the expression of surface ligands and receptors and can carry therapeutic cargo, both of which are determined by the parent cell and environmental conditions from which they originate. For example, exosomes derived from mesenchymal stem cells (MSCs) and cardiosphere-derived cells (CDCs) have been shown to possess cardioprotective effects (5–9). Furthermore, exosomes can be modified through loading of therapeutic cargo and their targeting abilities honed through surface protein modification.

Upon discovery that exosomes could retain a sensitive cargo and move unabated from one location within the body to another, their potential as a therapeutic delivery vehicle garnered much attention. Exosomes encompass many of the features of an ideal delivery vehicle, including a long circulation time, low levels of clearance and degradation, and preservation of the therapeutic activity of its cargo (10). Phase I clinical trials utilizing dendritic cell-derived EVs have demonstrated feasibility and short-term safety of autologous EV administration (Table I) (11–14). Going forward, a detailed understanding of their biogenesis, molecular composition, surface proteins, and biodistribution profile is crucial in maximizing the potential to engineer these vesicles into a clinically relevant delivery system.

## EXTRACELLULAR VESICLE NOMENCLATURE AND CLASSIFICATION

Extracellular vesicles can be broadly classified into three main groups based on their mode of biogenesis: (1) *exosomes*, small (30–100 nm) vesicles of endocytic origin; (2) *microvesicles*, also known as shedding vesicles, ectosomes, or microparticles—medium sized (50–1000 nm) particles shed directly from the plasma membrane; and (3) *apoptotic bodies*, larger (50–5000 nm) blebs released by dying cells. There is a great deal of discrepancy in nomenclature and purification criteria in the literature with a definitive categorization yet to be achieved (15). As such, a minimal set of biochemical, biophysical, and functional standards have recently been put forth by the International Society for Extracellular Vesicles (ISEV) (15). Interestingly, there appears to be heterogeneity even within subtypes, further complicating subpopulation classification (16,17).

## BIOGENESIS OF EXTRACELLULAR VESICLES

### Exosomes

**Fig. 1.**
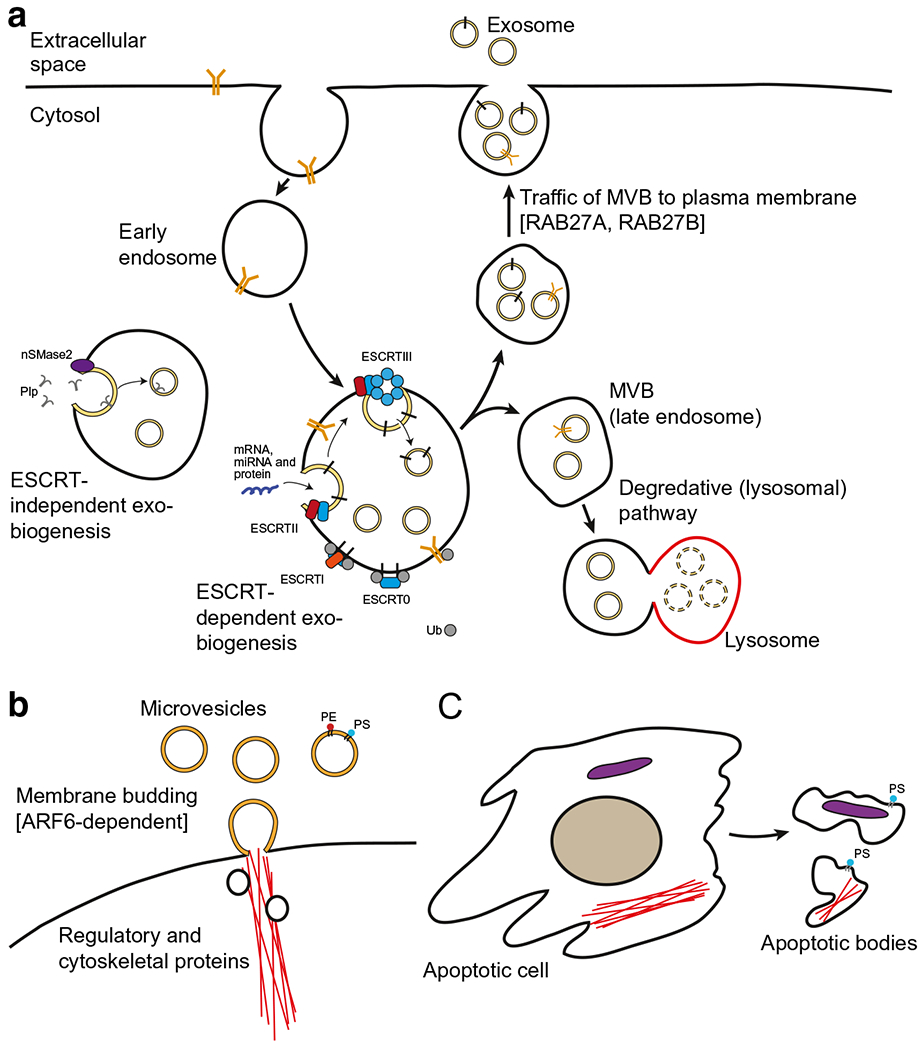
Biogenesis of EVs: exosomes, microvesicles and apoptotic bodies. **a** Exosomes originate from a double invagination of the plasma membrane. Their formation at endosomes is heavily dependent on ESCRT machinery. nSMase2 and members of the RAB GTPase family play different ESCRT-independent roles in exosome biogenesis. **a** *modified from Robbins et al*. **b** Microvesicles are derived from budding of the plasma membrane, controlled by regulatory and cytoskeletal proteins. Their membrane is comprised of homogenously distributed phosphatidylserine (PS) and phosphatidylethanolamine (PE). **c** Apoptosis results in the formation of apoptotic bodies. These vesicles are irregular in size and shape and contain nuclear fractions and cytoplasmic organelles along with extensive amounts of phosphatidylserine in their membrane. MVB, multivesicular bodies; Ub, ubiquitin

Exosomes are the most extensively researched sub-group of extracellular vesicles and are formed from the inward budding of endosomal membranes (Fig. 1a). When pinched off, these invaginations form intraluminal vesicles (ILVs). Endosomes containing ILVs are referred to as multivesicular bodies (MVBs). The MVBs can either fuse with the plasma membrane and release ILVs as exosomes, or complete the endolysosomal pathway with ILV digestion and degradation by lysosomes (18). It is still poorly understood why certain MVBs are sent to lysosomes for degradation and others fuse with the plasma membrane for release, but secreted MVBs appear to be richer in cholesterol and preferentially fuse with the plasma membrane (19,20).

The majority of exosome formation at endosomes is dependent on the endosomal sorting complex for transport (ESCRT) machinery, although a subset of proteins (e.g., PLP) are sorted into ILVs independently of ESCRTs through raft-based microdomains (21–23). The first ESCRT dependent event that takes place is the clustering of cargo for exosomal packaging. This is orchestrated by ESCRT-0 which recognizes ubiquinated proteins on the cytosolic side of the MVB. ESCRT-0 localizes at the surface of the MVB and recruits ESCRT-I in a process that is mediated by Vps27 complex (24). ESCRT-I then forms a complex with ESCRT-II and initiates membrane budding of the endosome. Additionally, the ESCRT-I-II complex brings the cargo retrieved by ESCRT-0 into the area of membrane budding, where it will eventually be packaged into an exosome. ESCRT-III is subsequently recruited to the site of budding, where it catalyzes membrane scission, effectively completing the process of membrane budding. ESCRT-III is also responsible for delocalization of the ESCRT complex from the MVB, as well as recruiting deubiquitinases to ensure that biomolecules packaged into exosomes are not modified.

Neutral spingomyelinase 2 (nSMase2) and the RAB family of small GTPase also play essential roles in exosome biogenesis through ESCRT-independent mechanisms (25). nSMase2 contributes to exosome secretion by triggering the budding of exosomes into MVBs. shRNA blockade of nSMase2 has been shown to inhibit release of exosomes from human macrophages (26) and human CDCs (9). In contrast, Rab27a and Rab27b control distinct aspects of MVB trafficking, docking, and fusion with the plasma membrane (27). Inhibition of Rab27a decreases secretion of a subset of exosomes bearing CD63, Hsp 70, Tsg101, and alix, but does not affect the secretion of vesicles carrying CD9 and Mfge8 (28). Silencing of effector proteins Slp4 and Slac2b phenotypically mimics silencing of Rab27 and Rab27b, respectively, highlighting their roles in MVB exocytosis and exosome secretion.

### Microvesicles

In contrast with exosomes, microvesicles are plasma membrane-derived particles released into the extrcellular space by outward budding and fission of the plasma membrane (Fig. 1b). The biogenesis cascade of microvesicles is controlled by regulatory and cytoskeletal proteins resulting in phospholipid redistribution and cytoskeletal protein contraction. Microvesicle formation is induced by translocation of phosphatidylserine to the outer-membrane leaflet through the activity of aminophospholipid translocases (29). The process of microvesicle budding is subsequently regulated by the GTP-binding protein, ADP-ribosylation factor 6 (ARF6). ARF6 is a mediator of cell recycling, notably active in macrophages during phagocytosis. In its activated form, ARF6 initiates a signaling cascade that starts with the activation of the enzyme phospholipase-D and ends with the phosphorylation and activation of myosin light-chain kinase (29). Extracellular signal-related kinase is then recruited to the plasma membrane where it activates myosin light-chain kinase, triggering the release of microvesicles.

### Apoptotic Bodies

As opposed to the heavily regulated biogenesis and release of exosomes and microvesicles, apoptotic bodies are formed as a result of programed cell death (Fig. 1c). This class of extracellular vesicles is a heterogeneous population with a variety of irregular shapes and sizes. As apoptosis beings, chromatin within the nucleus condenses and organelles begin to disintegrate. Shortly after, the cell membrane forms blebs, the cell shrinks, and the organelles brake down. The loose contents of the misshapen cell form the basis of the plasma membrane-bound vesicles known as apoptotic bodies (30).

## COMPOSITION OF EXTRACELLULAR VESICLES

Extracellular vesicles contain lipids, nucleic acids and proteins, the content of which varies with their mode of formation and cellular origin. In a similar manner, while the membranes of each subset of EVs form as a lipid bilayer, the composition of proteins and lipids differs between EV populations secondary to their biogenesis.

### Exosomes

There are currently over 92,897 protein entries, 27,642 mRNA entries, 4934 miRNA entries, and 584 lipid entries in Vesiclepedia associated with EVs (4). Within the subpopulations of EVs, exosomes comprise the majority of protein, miRNA, and lipid-based entries, highlighting their importance in cellular communication and recent interest within the scientific community. The composition of exosomes is not a mere reflection of the cell. Exosomes are enriched in specific proteins, lipids, and RNAs whereas others are absent, indicating the existence of specialized sorting mechanisms. Proteins enriched in exosomes include tetraspanins, integrins, immunoglobulins, and growth-factor receptors, cytoskeletal proteins (tubulin, actin), ESCRT-related proteins (Alix, Tsg101), heat-shock proteins, and proteins involved in vesicle trafficking such as Rab GTPases, annexins, and flotillin (31). The selection of exosomal cargo encapsulated into ILVs is a selectively regulated process that reflects both the nature of the parent cell as well as its pathophysiological state (32). For example, EVs derived from MSCs contain an array of pro-angiogenic proteins whereas EVs derived from antigen presenting cells contain MHC molecules enabling them to elicit an immune response (33,34). Tumor cell-derived exosomes have been shown to contain oncogenic proteins such as c-Met oncoprotein and TGF-β1, which promote angiogenesis and tumor-cell proliferation (35,36). Proteins can be recruited as a response to an environmental stimuli or stress. Exosomes derived from MSCs cultured under ischemic conditions are secreted in greater quantities and contain higher amounts of epithelial growth factor, fibroblast growth factor, and platelet-derived growth factor as compared with MSC-exosomes derived under physiological conditions (33). Exosomal proteins can also be packaged as a by-product of their biogenesis (37). For example, Alix and TSG101 function as accessory proteins in the ESCRT pathway, and are incorporated into all exosomes.

The selection of exosomal miRNA cargo also appears dependent on the parent cell and environmental factors. Some miRNA species, including miR-451a, are selectively enriched in EVs of multiple cell types (38). This sorting is highly regulated, the complexity of which is still unfolding. Recent studies indicate that specific miRNA motifs and their interaction with specific chaperon proteins facilitate incorporation into exosomes, as well as post-transcriptional modifications such as 3′ end adenylation and uridylation (39–41). Additionally, RNA-binding proteins, such as SYNCRIP, hnR NPA2B1, and Y-box protein 1, have been shown to mediate the exosomal sorting of miRNAs (39,42,43).

Exosomes are also heavily enriched in lipids, as evidenced by their unique rigid lipid bilayer membrane (44). While bearing the same orientation as the plasma membrane, the exosome membrane is not identical to its parent lipid bilayer, but instead selectively enriched in sphingolipids, cholesterol, glycerophospholipids, ceramide, teraspanins (CD63/Lamp3, CD81, CD9, CD82), membrane proteins associated with lipid rafts (glycosylphosphatidylinositol-anchored protein and flotillin), endosome-associated proteins (Alix and Tsg101), heat-shock proteins (HSP70, HSP90), and integrins (40,45,46). The exosome membrane is resistant to freeze-thaw cycles and facilitates efficient delivery to various cells. Sphingomyelin and monosialodihexosylganglioside (GM3) determine the rigidity of the lipid membrane, and phosphatidylserine facilitates signaling and fusion to the plasma membrane (47).

### Microvesicles

The composition of microvesicles depends largely on the cell type from which they originate (48). Microvesicles have been found to contain mRNA, noncoding RNA, cytosolic and membrane proteins such as oncogene and other growth-factor receptors, intergrins, MHC class I molecules, and soluble proteins such as proteases and cytokines. They also express CD40 ligand which in atherosclerotic lesions interacts with endothelial CD40 to promote *in vivo* angiogenesis, likely contributing to increased plaque vulnerability (49). GO analyses has demonstrated microvesicle enrichment in proteins normally associated with mitochondria, the endoplasmic reticulum, and proteasomes (50). Despite the internal cargo mimicking the cytosol of the cell, the membrane composition of microvesicles remains distinct from the parent cell with significant remodeling enabling specialized function. Not all plasma membrane proteins are incorporated into the microvesicles although the topology of the membrane proteins remains intact. The lipid composition of the microvesicle membrane lacks the asymmetric distribution characteristic of the plasma membrane, with aminophospholipids phosphatidylserine (PS) and -ethanolamine (PE) homogeneously distributed throughout the MV membrane upon formation (51). PS is relocated to the outer-membrane leaflet, specifically at sites on the cell surface where microvesicle shedding occurs. PS exposure provides a signal for recruitment of macrophages to bind to and engulf apoptotic cells (52).

### Apoptotic Bodies

Secondary to their biogenesis and in contrast to exosomes and microvesicles, apoptotic bodies contain nuclear fractions and cytoplasmic organelles. As early redistribution of plasma membrane phosphatidylserine is a general feature of apoptosis, a defining feature of apoptotic bodies is the extensive amounts of phosphatidylserine in their membrane.

## ISOLATION AND CHARACTERIZATION OF EXTRACELLULAR VESICLES

### EV Isolation

Extracellular vesicles can be isolated from biological fluids and cell culture supernatant using a wide variety of isolation techniques, as previously reviewed (53,54). Each method exploits various EV traits including size, shape, density, and surface receptors (55). Knowing that exosome isolation methods can influence EV integrity, biodistribution, and functional properties, it is important to consider the enrichment methods chosen for therapeutic applications (56,57). A combination of strategies may be optimal to yield the highest purity and most therapeutically effective fractions, as the field currently lacks a “gold standard” isolation method.

Differential ultracentrifugation (UC) is one of the most widely used EV isolation techniques, separating particles based on their sedimentation coefficients (58). Differential centrifugation can be followed by density gradient ultracentrifugation to separate low-density EVs from high-density protein aggregates that often contaminate EV ultracentrifugation pellets. Variations of this process include density gradient centrifugation (DGC), isopycnic centrifugation, and moving-zone centrifugation, all of which aim to improve upon the yield and purity of isolated EVs (55,59,60). Due to the significant overlap in densities and sedimentation coefficients between vesicles in biological samples, it is often necessary to perform sequential centrifugation isolations, varying the density and/or centrifugal force. This is a difficult isolation method to scale-up as ultracentrifugation is limited on input sample size (400 mL per run secondary to rotor sizes). Additional drawbacks to UC enrichment methods include vesicle aggregation and co-isolation of soluble factors, proteins, and ineffective fractions of EV aggregates, all of which can confound downstream applications (61).

Various commercial kits make use of volume-excluding polymers such as polyethylene glycol (PEG) and allow for rapid EV isolation from culture media or body fluids. Although easy to use and scale for larger isolations, these precipitation based methods lack isolation specificity, co-precipitating protein, polymeric materials, and other non-EV fractions (62). Additionally, as vesicles stay bound to the polymer post isolation, a post-processing step is needed to avoid interference with downstream analyses (this can be accomplished with a Sephadex G-25 column).

Ultrafiltration is another popular EV isolation technique to concentrate EV fractions from supernatant based on their size and molecular weight. The force required to push EVs through the membrane however, can result in deformation of larger particles which are subsequently forced through the filter and erroneously incorporated into downstream applications and analysis (55). Additionally, clogging and trapping of vesicles within membrane filters can reduce yield and efficiency of isolation.

Techniques that pair ultrafiltration with ultracentrifugation into a sucrose cushion or polyethylene glycol (PEG) have both been used to purify EVs for clinical application (63,64). Additional size-based EV isolation techniques include size exclusion chromatography (SEC), asymmetrical flow field-flow fractionation (AF4), label-free acoustic non-filter systems, and hydrostatic filtration dialysis (HFD) (65–68). While SEC allows separation of EV from the bulk of soluble proteins, contaminating particles in the EV size range such as lipoprotein complexes may be co-isolated as this method of separation is purely based on particle size. Isolation techniques relying on filtration are highly scalable and reproducible, with ultrafiltration and size-exclusion liquid chromatography-based methods appearing promising for large-scale EV bioprocessing (57).

Immunoaffinity capture, or immunoaffinity chromatography, utilizes the interactions of EV surface proteins with specific receptors or ligands. The method can yield pure EV subpopulations, but is highly influenced by both the choice of affinity reagent and the ligand density on different EV types (69). This technique will likely improve with advancements in unblocking antigens and elucidation of optimal EV tags (55).

Recent studies have directly compared different methods of isolation (DGC, UC, and commercially available precipitation kits) on EV-RNA yield and purity. DGC was found to yield the highest purity, albeit 3–8× less protein and fewer particles than the commercial kits, highlighting the tradeoff of yield vs. purity that must be considered when addressing a specific research question (70). Currently, combinations of techniques, such as density gradient centrifugation followed by size exclusion or immunoaffinity capture, are most commonly used (71).

Less conventional than the aforementioned techniques and focused on the ability to process small volumes, microfluidic systems have also been developed for the isolation of EVs from bodily fluids (72). This on-chip EV separation technology, based off the idea of capillary electrophoresis used for protein and DNA separations, combines isolation and analysis techniques into one portable, functional unit. Microfluidic techniques take advantage of the intrinsic mechanical and physical properties of EVs, including their size, shape, density, adhesive properties, and deformability. They have shown a higher EV recovery and purity when compared with conventional isolation methods, but harbor the challenge of improving throughput while retaining high particle sorting sensitivity. There are currently a number of microfluidics-based EV platforms for the detection breast, ovarian, bladder, and non-small cell lung cancer (73–76).

### EV Quantification and Characterization

It is crucial that extracellular vesicles are not only properly isolated, but also well characterized and quantified to enable accurate, interpretable, and comparable downstream analyses. While a number of EV quantification methods exist, there is currently a lack of consensus on the proper quantification (77). While our knowledge of exosome biogenesis has allowed the creation of genetic tools for modification of the secretome (9), the field remains limited by an inability to accurately assess exosomes at a single vesicle level.

Nanoparticle tracking analysis (NTA) is currently considered the best method available for exosome quantification. An analysis of Brownian motion via light scattering enables the quantification and size distribution of vesicles in a liquid suspension (78). By considering the particle-containing fluid density and temperature of the system, the diffusion coefficient and hydrodynamic radius are calculated. This tool allows for accurate measurements of particle size and concentration based on the inherent characteristics of those particles. As vesicles with similar Brownian motion cannot be distinguished from each other, this technique is commonly used in addition to other, more sensitive, characterization methods. Of note, the choice of camera level and detection threshold settings introduces potential variability, and should be standardized between samples and research groups.

While not a common tool for exosome quantification (secondary to exosome loss during dehydration and embedding), electron microscopy (EM) remains the gold standard for verifying the quality of EV preparations. Transmission and scanning electron microscopy are most frequently used in the analysis of EVs. SEM has been shown to be less-time consuming and able to achieve greater image resolution than TEM, enabling the optimal imaging EV native morphology (79).

In addition to NTA and EM, exosome quantification is commonly reported in terms of total extracellular vesicle protein concentration. This is typically achieved with sample sonication followed by a total protein assay, with the total protein content serving as a surrogate measure of EV quantity. Western blot, ELISA, and/or flow cytometry is subsequently performed to identify and quantify specific EV proteins. The utility of this method of quantification is questionable when comparing exosomes from different cell sources or donors, as normalized protein content may not always equate to normalized bioactive load. Continuing to understand the mechanism/s of action of EV-based therapeutics remains essential for development of suitable potency assays and eventual clinical translation, as EV dosage may likely differ between samples quantified as “equivocal” by the methods discussed above (80,81).

## BIODISTRIBUTION

Similar to synthetically designed nanoparticles, the biodistribution of extracellular vesicles influences their therapeutic efficacy as well as their offsite toxicity (82). Therefore, a detailed understanding of the *in vivo* fate and pharmacodynamic properties of extracellular vesicles including tissue distribution, blood levels, and urine clearance are paramount for future therapeutic applications. Most EV biodistribution studies are limited by their use of fluorescent and bioluminescent pre-labeled purified extracellular vesicles. It must be taken into consideration that each EV labeling technique has associated advantages and limitations and the method used should reflect the aims of the experiment (Table II). Small lipophilic fluorescent dyes, such as PKH67, DiD, and DiR, have been commonly used for *in vivo* tracking of EVs and incorporate into the membrane lipid bilayer of exosomes through selective partitioning. Perhaps most problematic is their prolonged *in vivo* half-life, which ranges from 5 to > 100 days. This may create a scenario whereby dye-labeled EVs may be degraded and/or recycled while the dyes themselves remain intact and visible in situ, yielding inaccurate spatiotemporal information regarding the fate of the EVs (88). In addition, these membrane stains are typically lost following aldehyde-mediated fixation and lipid extraction, limiting their usefulness in investigations involving downstream IHH and ICC (92). It is unknown if these dyes change the composition of the vesicular membrane bilayer, including the presence of specific target molecules in the membrane bilayer, and therefore affect the uptake or downstream function of labeled extracellular vesicles. To avoid the potentially confounding effects associated with staining EVs, additional EV membrane labeling methods have been generated which involve fusion of fluorescent markers (eGFP or tdTomato) to exosomal sequences (NH2-termini of palmitoylation (Palm) signal or CD63 tetraspanin) (84), as well as nucleic acid stains which label the endogenous RNA/DNA cargo (ExoGlow-RNA™, SYTO RNASelect™) (87). As Palm Nh2-termini has been shown to primarily label the inner EV membrane, this strategy results in minimal disturbance to EV surface molecules. In addition to fluorescent labeling, a more quantitative strategy for downstream pharmacokinetic analysis has been described that involves tagging EVs with a radiotracer such as biotin ^125^I-BB or ^99m^TC-HMPAO to monitor dynamic systemic distribution and organ uptake (85,86). Fluorescent and radiolabeling methods are still limited, however, in the discrimination of functional uptake of EVs (EV mRNA transfer and subsequent protein translation vs. lysosomal degradation of EV content). While not a quantitative measure of EV uptake, utilization of a Cre recombinase-based system (which would use a permanent genetic switch to label cells that have internalized EVs and translated Cre mRNA) would allow for assessment of physiological EV uptake *in vivo* (89–91).

**Fig. 2.**
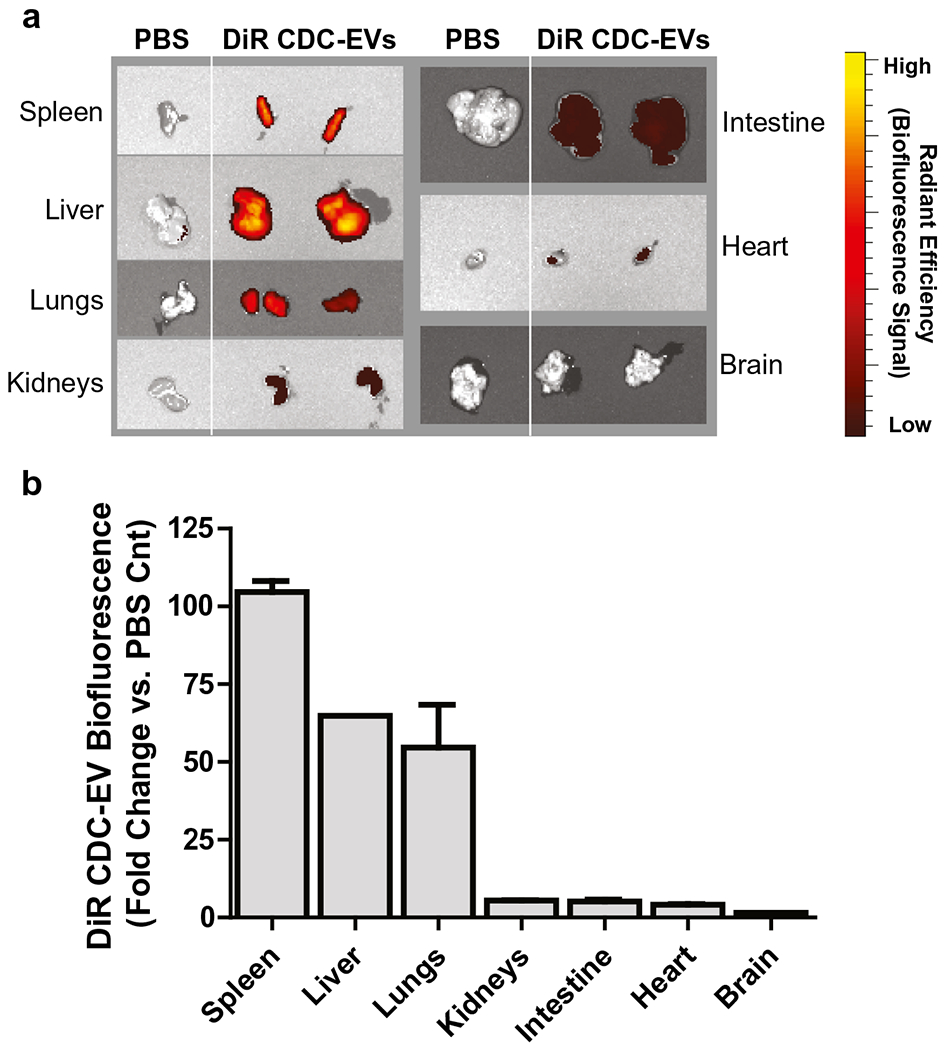
Biofluorescence of DiR labeled CDC-EVs. Human CDC-derived EVs were labeled with 1 μM DiR (Invitrogen) then washed with PBS by ultrafiltration to remove residual dye. Twelve-week-old C57BL/6 mice received a systemic injection of EVs or PBS control. **a** Representative IVIS images of organs (24 h post-injection) from mice injected with CDC-EVs or PBS. **b** Normalized biofluorescence signal in each organ expressed as a ratio of DiR CDC-EVs/PBS control. *N* = 4; Data expressed as mean ± SEM

It is widely reported that unmodified exosomes delivered through a systemic route preferentially accumulate in the liver, spleen and kidneys and are eliminated through biliary excretion, renal filtration, or the reticuloendothelial system (56,93). At 24 h post systemic injection, we found that DiR labeled CDC-derived EVs accumulated primarily in the spleen, followed by the liver and lung in high concentrations and the kidneys, intestines, heart and brain at a lower but still detectable level (Fig. 2). In a detailed biodistribution study involving DiR labeled exosomes from 4 different types of cells: HEK293T human embryonic kidney cells, murine B16F10 melanoma cells, C2C12 murine myoblast cells, and bone marrow-derived dendritic cells (DCs), Wiklander *et al.* also found that EVs largely distributed to organs of the mononuclear phagocyte system, with the highest accumulation in the liver, followed by the spleen, GI tract, and lungs (56). This finding is in line with recent work demonstrating that cells of the innate immune system facilitate sequestration and clearance of EVs upon introduction into the biological environment (94). Clearance of exosomes in macrophage depleted mice is significantly delayed compared to control animals, suggesting that macrophages play a pivotal role in the clearance of EVs from blood circulation irrespective of the EV cell of origin (95). Similar to macrophage recognition of apoptotic cells by phosphatidylserine (PS) on the outer leaflet of the plasma membrane (96), macrophages appear to recognize the negative charge of PS exposed on the surface of exosomes through the class A macrophage scavenger receptor (SR-A) (97).

Using an eloquent metabolic biotinylation and luciferase labeling system (EV-GlucB reporter), the biodistribution and clearance of systemically injected human HEK293T-derived EVs in mice was evaluated by *in vivo* and *ex vivo* analysis (88). EVs were detected predominantly in the spleen, followed by the liver, kidneys and lungs 30 min after IV injection. There was an initial fast distribution phase with a half-life of 20 min, followed by a longer elimination phase with a half-life of 180 min. The majority of EVs were subsequently cleared from the animals by 6-h post-injection, indicating active cellular uptake and degradation of the EVs. The difference in biodistribution between studies may be attributed to the cell type used as well as a variation in EV isolation methods, both of which dictate the shape, size, surface protein, lipid composition, and population of purified EVs. In addition, the high splenic uptake may be attributed to the administered EV dosage (100 μg) which in excess, may result in saturation of liver macrophages, higher free EV levels in the blood, and spillover into the splenic vasculature (98).

It is unclear the extent of which EV donor cell type affects subsequent biodistribution and clearance *in vivo*, however, studies suggest that EVs secreted by some cell types do exhibit target selection. For example, when comparing exosomes from C2C12 cells, bone marrow-derived DCs, and B16F10 melanoma cells (administered at the same dosage and consisting of the same EV size profile) DC-EVs had the highest distribution to the spleen (56). This suggests a natural tropism, possibly acquired from their parental cell of origin through expression of ICAM-1 on DC-EVs and interaction with lymphocyte function-associated antigen (LFA-1) expressed on T cells (99). Interestingly, while MSCs injected into a healthy mouse localized to the liver and spleen, those injected into a mouse model of acute kidney injury also accumulated in the kidney (100), similar to the honing capabilities of their parent cell which occur via CD44 and hyaluronic acid interactions (101). Given that cancer cells produce an abundant amount of EVs and can display oncogenic receptors such as EGFRvIII on their surface, cancer-derived EVs will likely exhibit altered circulation, biodistribution, and clearance properties from their normal counterparts, with additional changes associated with both disease progression and remission (102).

Varying the route of administration and scaling the dosage can also have an effect on EV biodistribution. While IV injected HEK293T-derived EVs were found to accumulate in the liver, spleen, lung, and gastrointestinal tract, changing the administration route to IP caused an accumulation of EVs in the liver, pancreas, and gastrointestinal tract, identifying a means for altering EV distribution to a particular tissue target (56). The same group looked at the effects of dose titration of DiR labeled HEK293T EVs and found that an increase in dose resulted in an increased fluorescent signal but a relative decrease in accumulation in the liver, possibly secondary to a saturation of the reticuloendothelial system (RES) and an effective bypass of the liver at higher doses (1.5 × 10^10^ particles/g body weight) (56). Other studies have also shown that high concentrations of EVs can lead to accumulation in the lungs and asphyxiation, and should be avoided during therapeutic delivery. Smyth *et al.* demonstrated that 400μg of 4T1-derived EVs injected intravenously resulted in aggregation of exosomes in the lungs and animal death, whereas lower IV dosages (60 μg) distributed to the liver and spleen without any of the observed side effects seen at the higher concentration (94). It appears that up to 150 μg of exosomes have been systemically administered in mouse models without pulmonary complications (83).

## EXTRACELLULAR VESICLE ENGINEERING STRATEGIES

The majority of extracellular vesicles show limited cellular tropism, with a few exceptions (99,103). This has prompted the development of several targeting strategies for systemically delivered EVs to enhance their therapeutic applicability. In addition, the methodology for loading EVs with non-native cargo continues to expand, further extending the therapeutic capabilities of extracellular vesicles. Current strategies can be largely grouped into two main categories: approaches that focus on cellular modification and those centered on direct extracellular vesicle alteration.

### Indirect EV Engineering *via* Parent Cell Modification

Cell engineering techniques such as genetic modification, metabolic labeling, and exogenous delivery have been shown to change the surface expression and cargo of extracellular vesicles (104). Manipulating these processes with EV functionality in mind has allowed for significant advancements in receptor systems and therapeutic function.

The proteins expressed on the surface of EVs are an integral variable in EV biodistribution and cell-targeting capabilities. Minor differences in exosomal tetraspanin-complexes have been shown to strongly influence target cell selection *in vitro* and *in vivo* (105). Modification of these surface proteins remains a heavily researched area with the goal of improved targeting of EVs to tissues and cells types of interest. One such approach is the use of cellular transgene expression to create a modified EV membrane protein with signaling or homing properties. This can be accomplished by inserting the coding sequence of the ligand of interest inframe to the coding sequences between the signal peptide and N-terminus of the mature peptide of a transmembrane protein. When this fusion cassette is expressed in cells using a gene transfer vector (such as a lentiviral vector), transduced parent cells generate exosomes expressing the peptide of interest on their surface. As previously discussed, this has been widely utilized for stable integration of fluorescent and luciferase fusion proteins within the exosomal membrane for in *in vivo* biodistribution and cell uptake studies (106).

In addition to creating reporter systems, this approach has been utilized to enhance EV therapeutic efficacy. Commonly modified transmembrane proteins include tetraspanins (CD63, CD9, CD81) (106), lysosome-associated membrane glycoprotein 2b (Lamp-2b) (107), glycosylphosphatidylinositol (GPI) (108), platelet-derived growth-factor receptors (PDGFRs) (109), and lactadhein (C1C2 domain) (110,111). Alvarez-Erviti *et al.* fused the rabies viral glycoprotein (RVG) with Lamp-2b to target exosomes to neurons and glia (107). Targeted exosomes were shown to cross the blood-brain barrier and deliver functional cargo (exogenously loaded siRNA), resulting in BACE1 knockdown. A similar fusion protein approach for exosomal targeting was employed by Tian *et al.*, who engineered immature dendritic cells to express Lamp2b fused to αv integrin-specific iRGD peptide (112). Purified exosomes were loaded with doxorubicin and demonstrated efficient targeting and drug delivery to tumor cells *in vitro* and *in vivo*. In a similar manner, Ohno *et al.* fused an EGFR specific binding peptide, GE11, to PDGFR and showed that systemically injected EV delivered let-7a miRNA to EGFR-expressing xenograft breast cancer tissue with a therapeutic response (113). Finally, Yim *et al.* recently described a unique optogenetic exosome system, termed EXPLORs (exosomes for protein loading via optically reversible protein-protein interactions), which utilized a CD9-CIBN fusion protein and CRY2-conjugated cargo proteins (114). Taking advantage of the protein’s blue light-dependent phosphorylation (115), cargo proteins introduced into exosomes via endogenous biogenesis were able to detach from CD9-conjugated CIBN with removal of the light source, resulting in their release into the exosomal intraluminal space and enabling their efficient delivery to the cytosolic compartment of target cells.

In addition to modifying extracellular vesicle membranes through genetic engineering of their parent cell, the therapeutic cargo of EV is also able to be manipulated by altering various aspects of their regulated biogenesis. In a recent study, Sterzenbach *et al.* used the evolutionarily conserved late-domain (l-domain) pathway as a mechanism for loading exogenous proteins into exosomes (91). Labeling target proteins with a WW tag led to recognition by the l-domain-containing protein Ndfip1, resulting in ubiquitination and packaging into exosomes.

Differing from the mechanisms regulating protein localization, cis-acting regulatory sequences (known as zipcodes) and trans-acting proteins are considered to be the main driving forces of mRNA localization and post-transcriptional regulation (116,117). Bolukbasi *et al.* described a consensus sequence present in the 3′UTRs of a number of mRNAs enriched in tumor-cell MVs which resulted in twofold mRNA enrichment in EVs, as compared to their cells of origin using a reporter mRNA (118). While this sequence may not be a universal mechanism, the prospect of identifying additional zipcode-like sequences able to target mRNAs to MVs is important in many different aspects of MV dynamics, and opens the door for engineering mechanisms to load mRNAs. Recently, Hung *et al.* described a platform for actively loading engineered cargo RNAs into EVs, referred to as the Targeted and Modular EV-Loading (TAMEL) approach (119). They found that while active loading of mRNA-length (> 1.5 kb) cargo molecules was possible, loading was significantly more efficient for smaller (~ 0.5 kb) RNA molecules. Despite high EV-loading efficiencies and substantial EV uptake by recipient cells, most cargo was rapidly degraded in recipient cells. This was primarily due to the rapid degradation of the EV cargo upon internalization in the recipient cells, highlighting the inefficient endosomal escape when taken up by the recipient cell lines. As this study used HEK293T-derived EVs, it would be informative to explore engineering platforms in other donor cells such as MSCs, CDCs, or dendritic cells.

The process of selectively loading miRNA appears to be more complicated, as RNA-binding proteins required for miRNA sorting have been shown to vary between cell types (43). Additionally, multiple RNA-binding proteins are involved in miRNA sorting mechanisms, making it more complicated to understand the cascade of events that takes place when miRNAs are specifically sorted into exosomes (43). In T cells, miRNA sorting into exosomes begins with sumoylation of the heterogeneous nuclear ribonucleoprotein A2B1 (hnRNPA2B1) (120). hnRNPA2B1 then binds to specific miRNAs and carries them to the surface of a multivesicular body where they are endocytosed and packaged into exosomes. Short sequence motifs, termed EXOmotifs and CLmotifs (40), determine the fate of the miRNA. In the case of miRNA containing an EXOmotif, the sumoylated hnRNPA2B1 is able to recognize and specifically bind the sequence motif exhibited by the miRNA. In the case of a miRNA with a CLmotif, the hnRNPA2B1 will not recognize the motif and, thus, will not bind to the miRNA. One strategy for passive endogenous loading of therapeutic nucleic acid cargo within EVs is to transfect oligonucleotides (miRNA/siRNAs/mRNAs) or a plasmid expressing the oligonucleotides of interest directly into the exosome producing parent cell (121). Several studies have shown that miRNAs can be efficiently loaded into EVs either via miRNA expression backbones or transfection of precursor or miRNA mimic/antimiR oligonucleotides (122,123).

### Direct EV Modification

In contrast to cellular modification strategies, directly encapsulating cargo into purified exosomes provides an alternate avenue for EV engineering that can avoid the inefficient incorporation seen with some cell-based technologies. There are two major approaches utilized in the incorporation of exogenous therapeutic agents into EVs—passive and active encapsulation. While passive-loading methods rely on spontaneous membrane interactions, active-loading strategies require the use of techniques that temporarily disrupt the EV membrane to allow influx of cargo. The encapsulation of hydrophobic drugs into EVs by physical entrapment is one example of passive EV loading. Sun *et al.* demonstrated that curcumin, a polyphenol with anti-inflammatory and antineoplastic activity, is self-assembled into the lipid bilayer of exosomes via hydrophobic interactions, resulting in increased longevity of the drug (124). The approach of hydrophobic sequestration is also used with many commercially available membrane dyes, such as DiI and PKH-67. Unlike the plasma membrane of their parent cells, the rigid bilayer of the EV membrane prevents spontaneous fusion with larger lipid-based particles, such as liposomes, under physiological conditions, requiring aggressive freeze-thaw cycles for membrane disruption.

Another passive strategy involves the utilization of multivalent electrostatic interactions to bind cationic species to the surface of negatively-charged EV membranes. Nakase and Futaki demonstrated this technique in exosomes by using cationic lipids and a pH-sensitive fusogenic peptide to enhance the cytosolic release of exosomal contents (125). The longevity of the released cationic materials are questionable however, as they are preferentially taken up by endocytosis and undergo subsequent lysosomal degradation (126).

Active EV-loading methods demonstrate higher loading efficiency than passive strategies. Sonication, one active approach, utilizes a homogenizer probe to shear the EV membrane and allow drugs to diffuse through newly created pores. Electroporation also aims to transiently permeabilize the EV membrane through the use of an applied electrical field on EVs in an electrolytic solution. However, as both techniques can result in EV membrane instability secondary to their disruptive approach, EVs need to be re-characterized post-loading to ensure retention of their therapeutic efficacy. Interestingly, permeabilization with saponin (a surfactant that generates pores in the membrane through its interaction with cholesterol) has been shown to result in enhanced bioactivity of EV-loaded agents when compared with sonication, possibly secondary to a higher degree of intact membrane proteins which results in more uniformity of their surface morphology and subsequent decreased clearance by macrophages (127).

To enhance cellular uptake of EVs, Nakase *et al.* used active EV engineering to modify EV membranes. Expression of modified arginine-rich cell-penetrating peptides resulted in activation of the macropinocytosis pathway, causing an increase in cellular EV uptake (128). Additionally, active EV modification can be used to modify circulation clearance and improve targeted uptake. Kooijmans *et al.* conjugated EVs with polyethylene glycol (PEG) to enhance their circulation time and prolong exposure to their target-specific receptor (129). Following an intravenous injection, they found that unmodified EVs are cleared from the circulation within 10 min, while exosomes modified with PEG remained in the bloodstream for over 60 min post-injection. As PEG shields EVs from interacting with plasma proteins, incorporation of PEG-conjugated nanobodies (also known as single-domain antibodies) allowed for enhanced tumor cell-targeting interactions (130).

### Extracellular Vesicle Mimetics

An alternative approach to engineering EVs is creating synthetic analogs which mimic the characteristics of endogenous EVs. EV mimetics allow for custom cargo selection and a scalable, well-characterized drug delivery system. Jang *et al.* generated doxorubicin-loaded EV-mimetic nanovesicles *via* serial extrusion of human U937 monocytes through polycarbonate membranes in the presence of doxorubicin (131). Following IV injection, the nanovesicles demonstrated improved targeting and delivery of chemotherapeutic drugs to the tumor site of CT26 mice when compared with systemically administered doxorubicin. Following the same method of serial extrusion, Oh *et al.* generated EV-mimetic nanovesicles from a murine pancreatic β-cell line to investigate their therapeutic potential in a diabetic immunocompromised mouse model (132). Treatment with EV-mimetic nanovesicles resulted in *in vivo* differentiation of insulin producing cells and maintenance of physiological glucose levels. In contrast to the serial extrusion method of generating nanovesicles, Sato *et al.* developed a two-step protocol for producing hybrid exosome-liposome constructs that combine the advantageous properties of EVs with the customizability of liposomes (133). They found cellular uptake of exosome-liposome hybrids to be nearly twofold higher than unmodified exosomes. This was attributed to the membrane modifications made possible by incorporation of liposomes into the hybrid construct.

## CURRENT APPLICATIONS OF ENGINEERED EVS IN DISEASE THERAPY

Both endogenous and engineered EVs hold tremendous promise as therapeutic tools for a wide variety of disease including cancer, diabetes, cardiovascular disease, and neurological disorders. Encouragingly, a number of EV phase I trials have demonstrated efficacy and feasibility in patients with cancer and diabetes (Table I). Perhaps the most significant progress however, can be gauged by the success of a number of preclinical studies which have utilized EVs both as drug delivery vehicles as well as carriers of de novo therapeutic cargo (Table III).

### Cancer

Cancer remains one of the leading causes of mortality worldwide secondary to metastasis and the development of multiple drug resistance (MDR). The potential to deploy targeted EVs with specialized payloads has shown potential for circumventing the very limitations of current radiotherapy and chemotherapy. For example, Hadla *et al.* demonstrated that higher intracellular concentrations of doxorubicin (DOX) can be achieved in breast and ovarian tumors when the chemotherapeutic agent is loaded into and delivered by EVs as compared to administered systemically. They also found a reduction in cardiotoxicity secondary to decreased crossing of DOX through the myocardial endothelium (134). In a similar manner, macrophage EV-encapsulated Paclitaxel demonstrated an increased neoplastic tropism and cytotoxicity in MDR pulmonary metastases (135).

In addition to their use in chemotherapeutics, numerous studies have also focused on exploiting the antineoplastic potential of their *de novo* miRNA cargo. O’Brien *et al.* found that loss of miR-134 in cells and their EVs was associated with increased cellular aggressiveness (136). They subsequently created miR-134-enriched EVs from a miR-134-transfected triple-negative breast cancer cell line and found the modified EVs reduced aggressiveness of secondary cells (via down-regulation of STAT5B-Hsp90) and increased sensitivity to anti-Hsp90 drugs. Overexpression of miR-122 in adipose-derived mesenchymal stem cell exosomes also resulted in inhibition of carcinoma growth and increased sensitivity to chemotherapy in xenograft mice (123). Similarly, in a rat model of glioma, marrow stromal cell-derived miR-146b-enriched exosomes were shown to silence EGFR and inhibit proliferation of tumor cells (137). In addition to miRNA, exosomes have also been shown to effectively deliver siRNA (exogenous double-stranded RNA) into target cells. Uptake of RAD51 and RAS52 siRNA enriched exosomes resulted in the reproductive cell death of fibrosarcoma cells *in vitro*, providing additional evidence of the ability to use EVs as vectors in RNAi-based gene therapy (138).

### Diabetes Mellitus

Diabetes (DM) is characterized by a resistance to insulin (type II) or the inability to produce insulin (type 1) and associated with end organ damage and life-threatening complications. More than one quarter of patients with diabetes will suffer from diabetic nephropathy which carries a significant increase in renal failure and mortality. Jiang *et al.* tested the therapeutic potential of human urinary stem cell exosomes in a streptozotocin-induced diabetic nephropathy rat model (140). Following weekly IV EV injections, they found reductions in urinary albumin and podocyte apoptosis as well as an increase in proliferation of glomerular endothelial cells, suggesting EVs may be a novel approach in the treatment of diabetic nephropathy. Diabetes is also associated with an increased risk of CNS damage, often resulting from an ischemic stroke. Venkat *et al.* administered bone marrow stem cell-derived exosomes to type II DM rats following a transient middle cerebral artery occlusion to investigate their potential for neurorestorative effects (141). Compared to a PBS control, they noted significant improvements in function outcome, a reduction in blood-brain barrier (BBB) leakage and hemorrhage, and an increase in axon and myelin density. In diabetic cognitively impaired animals, mesenchymal stem cell-derived exosomes were also shown to improve cognition through reparation of oxidative damage in neurons and astrocytes (142).

### Cardiovascular Disease

Cardiovascular disease (CVD) encompasses a group of disorders affecting the heart and vasculature, the most common of which is coronary artery disease. Current treatment, however, is limited in its regenerative potential. Preclinical cell therapy trials failed to demonstrate any significant engraftment or progenitor differentiation into new myocardium, but instead highlighted EVs as the key beneficial mediator of cell therapy. Work directed at understanding the cardiovascular bioactivity of various EVs has exploded in recent years with the goal of developing a novel means of cell-free cell therapy for heart disease.

Lai *et al.* first identified EVs in the conditioned media of MSCs and demonstrated their therapeutic valve in reducing infarct size in a myocardial ischemia/reperfusion (I/R) model (5). This subsequently laid the foundation for application in a number of disease models and further ignited interest in the therapeutic role of EVs in CVD. Arslan *et al.* demonstrated a similar reduction in infarct size in a model of I/R following administered of MSC-derived exosomes 5 min prior to reperfusion (6). GATA-4 overexpressing MSCs-derived exosomes were shown to contribute to increased cardiomyocyte survival, reduced cardiomyocyte apoptosis, and preserved mitochondrial membrane potential in cardiomyocytes cultured in a hypoxic environment (8). Exosomes derived from other cell populations, such as cardiac progenitor cells, have also shown promise in treatment of CVD. Chen *et al.* showed that CPC-derived exosomes enriched in miR-451/144 promoted cardioprotection by increasing cardiomyocyte survival *in vivo* in a model of I/R and H9c2 survial in vitro (143). Exosomes from cardiosphere-derived cells (CDCs) have also been shown to recapitulate the therapeutic effects of CDCs, largely mediated (though not completely replicated) by miR-146a (7).

### Neurological Disorders

EVs represent a promising therapy for a number of neurological disorders based on their “immune privileged” status and ability to penetrate the BBB (127). Several studies have utilized EVs as vehicles for the delivery of drugs and exogenous siRNAs (107,139). In a model of glioblastoma, intranasal delivery of exosome-encapsulated curcumin demonstrated rapid delivery to the brain, selectively uptake by microglia, and subsequently apoptosis (139). In a model of Parkinson’s disease, intranasal delivery of macrophage-derived catalase-loaded exosomes penetrated the BBB, significantly decreased brain inflammation, and improved neuronal survival (127). Systemic administration of MSC-derived EVs has been shown to improve motor coordination and enhanced neurogenesis in a traumatic brain injury mouse model (144). The de novo contents of EVs have also been explored for the treatment of neurological disorders. Xin *et al.* engineered MSC-derived exosomes to carry increased levels of miR-133b in an effort to enhance brain remodeling after stroke (145). IV administration of miR-133b-loaded exosomes showed improved functional recovery with increased axonal plasticity and neurite remodeling in the ischemic border zone in a rat model of middle cerebral artery occlusion.

## CONCLUSION

Extracellular vesicles hold great promise for use as therapeutic delivery vectors in disease. As natural mediators of intracellular communication, EVs are integral to numerous biological processes including repair and regeneration, resolution of inflammation, and tissue remodeling. Many studies have shown that EVs function as paracrine effectors, mediating a large degree of the benefits of cell therapy while eliminating many of the risks and limitations associated with cell engraftment and proliferation. Furthermore, due to their low immunogenicity, stability and high delivery capacity, EVs embody the definition of an ideal therapeutic delivery vehicle. They represent attractive nanocarriers for drugs as well as therapeutic small molecules, nucleic acids and proteins. These distinct advantages, together with our rapidly expanding ability to engineer EV cargo and surface marker expression for cell specific targeting, elevates their potential for future therapeutic success.

### Remaining Challenges

Several hurdles still lie on the horizon which must be addressed prior to successful clinical translation. Fully realizing the therapeutic potential of EVs requires standardization of our methodology for isolation, quantification, and characterization. EVs produced from different cell types are markedly different; even EVs secreted from the same cell type can vary in shape, size, and cargo secondary to donor-to-donor variability and differences in cell culture conditions. To complicate matters further, certain populations of cells appears to generate exosomes with multiple subtypes (81,146). This raises the hypothesis that a discrete subclass (referred to by Willis *et al.* as “signalosomes”) is responsible for their therapeutic potency (81). While we have made progress on genetic modification of the secretome, we are currently limited by our inability to isolate and characterize exosomes at the single vesicle level (9,81). Development of an exosome potency assay would be a valuable tool in overcoming exosome variations between sample preparations (81). Until this is developed, there will continue to be inconsistencies and debate over expressing EV dosage. Where possible, multiple quantification tools should be used to measure exosome concentration. To enable comparisons between cell type and reduce interoperator variability, all *in vitro* experiments and preclinical studies should report the administered EV dose as the quantity of EVs as well as the amount of cargo injected/added (expressed as the number of vesicle particles, amount of vesicle protein, and vesicle number to protein ratio). Furthermore, in engineered exosomes loaded with therapeutic cargo, loading efficiency should be expressed as both a percentage (cargo loaded into EV/cargo exposed to EV) as well as the number of therapeutic molecules/copies of loaded EV, to enable comparisons across different studies.

Future studies on the potential correlation of EV size, dosage, and pharmacokinetic properties will provide additional insights into EV-mediated therapies. It would be useful to employ high sensitivity methods of exosome tracking to compare *in vivo* properties of both exogenously and endogenously released EV from different cell types, isolated by different methods, and genetically engineered to express different surface receptors (88). Additionally, translating therapy from the lab to the clinic demands the ability to scale-up EV isolation for large-scale production. Label-free techniques are needed that can distinguish between EV subtypes with minimal sample variation and contaminants, as well as little to no disruption of EV integrity and potency.

### Future Perspectives

Engineered EVs provide a relevant and exciting therapeutic tool for the treatment of a variety of diseases ranging from cancer to Parkinson’s to ischemic cardiomyopathy. Research in the field of EVs is currently advancing at an exponential rate. As additional engineering techniques are developed and applied to improve their ability as functional carriers, we believe the prospect of harnessing EVs as a clinically relevant therapy in the next decade will become a reality.

## Figures and Tables

**Table I.** Human Clinical Trials Utilizing Exosomes as a Primary Therapy

Phase	Status	Disease	EV cellular origin	EV cargo	Reference
I	Completed; feasible and safe	Melanoma	Dendritic cells	Antigenic peptides	Escudier *et al.* (11)
I	Completed; feasible and safe	Non-small lung cancer	Dendritic cells	Antigenic peptides	Morse *et al.* (12)
I	Completed; feasible and safe	Colon cancer	Autologous ascites	Endogenous	Dai *et al.* (13)
II	Completed; feasible and safe	Advanced non-small lung cancer	IFN-γ-matured dendritic Cells	Antigenic peptides	Besse *et al.* (14)
I	Ongoing	Cutaneous ulcers	Plasma	Endogenous	NCT02565264
I	Ongoing	Colon cancer	Plant	Curcumin	NCT01294072
I	Ongoing	Type 1 diabetes mellitus	Mesenchymal stem cells	Endogenous	NCT02138331

Human clinical trials utilizing exosomes as a primary therapy

Studies obtained from *Clinicaltrials.gov*

**Table II.** Techniques for Labeling Exosomes

Labeling technique	Binding site(s)	Advantages	Limitations	References
(1) Membrane-bound Dye (e.g., DiR, DiD, PKH2,26,67)	EV membrane (selective partitioning)	→ Ease of labeling → Inexpensive	→ Low sensitivity and quantitative capacity → Inaccurate spatiotemporal information of EV fate (2° to long half-life) → Unable to use with IHC/ICC (signal lost post fixation and lipid extraction)	Peinado *et al.* (83)
(2) Fusion of fluorescent markers (e.g., eGFP, tdTomato) to exosomal sequences (e.g., Palm, CD63)	Inner and outer EV membrane	→ Stable expression → Cell type specific	→ Requires genetic modification → Low quantitative capacity (luminescent signal reduces with time)	Lai *et al.*, (84)
(3) Radiotracer (e.g., fusion protein of SAV-LA and radiolabeled ^125^I-IBB, ^99m^TC-HMPAO)	EV membrane (fusion protein), EV lumen (HMPAO)	→ Highly quantitative → Stable → Used clinically (HMPAO)	→ More time intensive → Analysis involves access and familiarity with radioactivity-based detection methods (^125^I-IBB) and/or SPECT/CT(^99m^TC-HMPAO) → Loss of EVs during labeling and separation protocols	Hwang *et al.* (85) Morishita *et al.* (86)
(4) EV nucleic acid stain and protein stain (e.g. ExoGlow™-RNA (System Biosciences), SYTO RNASelect™ (ThermoFisher), ExoGlow™-Protein (System Biosciences))	Internal EV mRNA or protein cargo	→ Ease of labeling → Observe transfer of EV contents → Ideal for in vitro uptake studies with live cell imaging → Low intrinsic background	→ Fluorescence signal lost with Formalin/PFO fixation on target cells for ICC/IHC (RNA stain) → Transient signal following EV cellular uptake (<1 week) → Limited use for *in vivo* biodistribution	Singh *et al.* (87)
(5) Fusion of membrane-bound variant of the Gluc reporter and biotin acceptor peptide (BAP, GlucB)	EV Membrane	→ Accurate spatiotemporal tracking *in vivo* and *ex vivo* → Allows for multimodal imaging (bioluminescence and fluorescence)	→ Requires genetic modification → More time intensive	Lai *et al.* (88)
(6) Cre recombinase-based system	EV lumen	→ Accurate assessment of physiological uptake *in vivo*	→ Not quantitative → More time intensive → Requires genetic modification	Fruhbeis *et al.* (89) Zomer *et al.* (90) Sterzenbach *et al.* (91)

*EV*, extracellular vesicle; *SAV*, streptavidin; *^125^I*, iodine-125; *LA*, lactadherin (an exosome-tropic protein); *HMPAO*, hexamethylpropyleneamineoxime; *PFO*, paraformaldehyde

**Table III.** Preclinical studies: EV Bioactivity and Therapeutic Implications

Disease	Cellular origin of EVs	EV cargo	Bioactivity	Potential therapeutic implications	References
Cancer	Multiple cancer cell lines	Doxorubicin	Increased therapeutic index, reduced off-target cardiotoxicity	Targeted cancer therapy, chemotherapy-induced cardiomyopathy	Hadla *et al.* (134)
	Macrophages	Paclitaxel, AA-PEG	Improved targeting to neoplasm, inhibited metastases growth	Pulmonary metastases	Kim *et al.* (135)
	Breast cancer cell line	miR-134	Reduced levels of Hsp90, reduced cancer cell migration and invasion	Triple-negative breast cancer	O’Brien *et al.* (136)
	Marrow stromal cells	miR-146b	Silenced EGFR, inhibited proliferation of glioma cells	Glioma	Katakowski *et al.* (137)
	HeLa cells / Ascites	RAD51 and R A D 5 2 siRNA	Reproductive cell death of fibrosarcoma cells	Fibrosarcoma	Shtam *et al.* (138)
	EL-4 cells	Curcumin	Induced apoptosis in microglia	Glioblastoma	Zhuang *et al.* (139)
	Adipose-derived MSCs	miR-122	Inhibited carcinoma growth, increased sensitivity to chemotherapy	Hepatocellular carcinoma	Lou *et al.* (123)
Diabetes	Urinary stem cells	Endogenous	Reduced urinary albumin and podocyte apoptosis, increased proliferation of glomerular endothelial cells	Diabetic nephropathy	Jiang *et al.* (140)
	Bone marrow stromal cells from rats with type 1 diabetes	Endogenous	Improved neurological functional outcome, increased axon and myelin density	Ischemic stroke in diabetics	Venkat *et al.* (141)
	Mesenchymal stem cells	Endogenous	Repaired oxidative damage in neurons and astrocytes	Cognitive impairment in diabetics	Nakano *et al.* (142)
Cardiovascular disease	ESC-derived MSCs	Endogenous	Reduced infarct size	Myocardial infarction, heart failure	Lai *et al.* (5)
	Mesenchymal stem cells	Endogenous	Reduced infarct size, increased ATP levels, decreased oxidative stress	Myocardial infarction, heart failure	Arslan *et al.* (6)
	Mesenchymal stem cells	GATA-4	Reduced infarct size, reduced cardiomyocyte apoptosis, preserved mitochondrial membrane potential	Myocardial infarction, heart failure	Yu *et al.* (8)
	Cardiac progenitor cells	Endogenous	Inhibited cardiomyocyte apoptosis	Myocardial infarction, heart failure	Chen *et al.* (143)
	Cardiosphere-derived cells	Endogenous	Mimicked CDC benefits	Myocardial infarction, heart failure	Ibrahim *et al.* (7)
Neurological disease	Dendritic cells	siRNA	Significantly reduced BACE1 gene expression	Alzheimer’s disease	Alvarez-Erviti *et al.* (107)
	Macrophages	Catalase	Improved neuronal survival, decreased brain inflammation	Parkinson’s disease	Haney *et al.* (127)
	Mesenchymal stem cells	Endogenous	Enhanced neurogenesis, improved motor coordination	Stroke, TBI	Doeppner *et al.* (144)
	Mesenchymal stem cells	miR-133b	Enhanced axonal remodeling and neurological function	Stroke, TBI	Xin *et al.* (145)

*ESCs*, embryonic stem cells; *MSCs*, mesenchymal stem cells; *TBI*, traumatic brain injury; *CDC* cardiosphere-derived cells
